# Deoxyshikonin-Induced Gene Expression Profile in Porcine Epithelial Cells

**DOI:** 10.3389/fvets.2021.711721

**Published:** 2022-01-13

**Authors:** Jing Wang, Wei Zhang, Xu Chu, Sixin Wang, Yamin Wang, Haifeng Ji

**Affiliations:** ^1^Institute of Animal Husbandry and Veterinary Medicine, Beijing Academy of Agriculture and Forestry Sciences, Beijing, China; ^2^Sino-US Joint Laboratory of Animal Science, Beijing Academy of Agriculture and Forestry Sciences, Beijing, China; ^3^College of Agriculture and Animal Husbandry, Qinghai University, Xining, China

**Keywords:** differential gene expression, immunity, natural compound, porcine epithelial cells, RNA-sequencing, transcriptome

## Introduction

Antibiotic feed additives are gradually being phased out in livestock production worldwide. There is an urgent need to develop novel antibiotic alternatives to ensure the health of livestock animals, and improving animal innate immunity through feed additives is one possible strategy to achieve this (1, 2). Natural products that have anti-inflammatory, anti-oxidative, and antibacterial effects have shown promising potential for health promotion and disease prevention (3). These are less likely to trigger bacterial resistance, and therefore are preferred as feed additives and potential antibiotic alternatives. Approximately 49% of the drugs approved by the U.S. Food and Drug Administration since the 1940s are natural products or their derivatives (4). Thus, the development of natural, active, classified green feed additives in the context of anti-resistance has broad application prospects.

Deoxyshikonin, an active component of *Lithospermum erythrorhizon*, is a promising drug candidate for the treatment of wounds and cancers (5, 6). In a screening of a library of 1,261 natural compounds using a newly established IPEC-J2/*pBD3*-luc cell line, we previously found that deoxyshikonin induces the expression of porcine host defense peptides (HDPs) (7). In addition, deoxyshikonin had a minimal effect on the expression of several representative inflammatory cytokine genes and obviously enhanced the antibacterial activity of 3D4/31 macrophages against both gram-negative and gram-positive bacteria. However, the underlying mechanism and biological functions of deoxyshikonin in porcine cells remained to be unraveled. Moreover, whether deoxyshikonin has a preventative effect on infectious diseases in animals requires further investigation. Therefore, in this study, we profiled deoxyshikonin-induced gene expression in porcine epithelial cells by RNA-sequencing (RNA-seq) with the aim to provide valuable clues for the in-depth study of deoxyshikonin.

## Methods

### Cell Lines and Culture Conditions

IPEC-J2 porcine intestinal epithelial cells were cultured in complete DMEM/F12 medium at 37°C in an atmosphere of 5% CO_2_ and 95% air, with 90% humidity. Complete DMEM/F12 medium is a 1:1 mixture of Dulbecco's modified Eagle's medium and Ham's F-12 (Gibco™, Thermo Fisher Scientific, Waltham, MA, USA) supplemented with 10% fetal bovine serum (FBS, Gibco™), streptomycin (100 μg/mL), penicillin (100 U/mL), and 1% ITS premix (5 μg/mL insulin, 5 μg/mL transferrin, and 5 ng/mL selenium) (ScienCell, San Diego, CA, USA). The cells were subcultured in complete medium every 2–3 days.

### Treatment of Cells With Deoxyshikonin

IPEC-J2 cells were seeded in six-well tissue-culture plates (Costar, Corning Inc., Corning, NY, USA) at 2.5 × 10^5^ cells/well. After overnight culture, the cells were treated with deoxyshikonin in triplicate at 5 μmol/L for 6 h. Non-treated cells served as a control. After incubation, the cells were washed with PBS three times and collected for further assays.

### RNA Extraction, Library Preparation, and Sequencing

Total RNA was extracted from the cells using RNAzol (Molecular Research Center, Cincinnati, OH, USA) according to the manufacturer's instructions. RNA integrity was determined using a 2100 Bioanalyzer (Agilent Technologies, Santa Clara, CA, USA), and the RNA was quantified using an ND-2000 instrument (NanoDrop Technologies). Only high-quality RNA samples (RIN ≥ 6.5, OD260/280 = 1.8–2.2, OD260/230 ≥ 2.0) were used for sequencing library construction. The RNA-seq libraries were prepared using the TruSeq™ RNA Sample Prep Kit (Illumina, San Diego, CA, USA) per the manufacturer's instructions. The libraries were size-selected for cDNA target fragments of 300 bp on 2% Low-Range Ultra Agarose, followed by PCR amplification using Phusion DNA polymerase. After quantification using a TBS380 fluorometer (Turner BioSystems, Sunnyvale, CA, USA), the libraries were sequenced in paired-end mode (2 × 150 bp) using an Illumina HiSeq X Ten or NovaSeq 6000 sequencer.

### Genome Alignment and Gene Annotation

The raw, paired-end reads were trimmed and quality-controlled using SeqPrep (https://github.com/jstjohn/SeqPrep) and Sickle (https://github.com/najoshi/sickle) with default parameter settings. The clean reads were aligned to the pig reference genome Sscrofa11 (http://asia.ensembl.org/Sus_scrofa/Info/Index) using TopHat v2.1.1 (http://ccb.jhu.edu/software/tophat/index.shtml) (8). For each sample, the mapped reads were assembled into transcripts using StringTie (https://ccb.jhu.edu/software/stringtie/) (9). Genes were annotated by BLAST searches against the Clusters of Orthologous Groups (COG), Gene Ontology (GO), and Kyoto Encyclopedia of Genes and Genomes (KEGG) databases.

### Analysis of Gene Expression Levels and Identification of Differentially Expressed Genes

Gene expression was quantified and normalized to fragments per kilobase per million reads (FPKM) using RSEM v1.3.1 (http://deweylab.biostat.wisc.edu/rsem/). Differential expression was analyzed using DESeq2 v1.24.0 (http://bioconductor.org/packages/stats/bioc/DESeq2/) (10). *P*-values were adjusted using the Benjamini and Hochberg approach to control the false-discovery rate. Genes with an adjusted *P* < 0.05 and fold change (FC) ≥ 2 (log_2_FC ≥ 1) were considered significantly differentially expressed.

### Functional Enrichment Analysis of DEGs

To assess DEG functions, the DEGs were subjected to GO enrichment analysis based on Fisher's exact test, using Goatools v0.6.5 (https://github.com/tanghaibao/GOatools). KEGG pathway enrichment analysis was carried out using KOBAS v2.1.1 (http://kobas.cbi.pku.edu.cn/download.php) to assess significantly enriched metabolic or signal transduction pathways (11). *P*-values were adjusted using Bonferroni correction, and GO terms and KEGG pathways with adjusted values of *P* < 0.05 were considered significantly enriched.

### Validation of DEGs by Reverse-Transcription Quantitative PCR

Eight DEGs were selected for validation by RT-qPCR using the extracted total RNA. One microgram of total RNA was reverse-transcribed into cDNA using an iScript™ cDNA Synthesis Kit (Bio-Rad, Hercules, CA, USA) according to the manufacturer's instructions. qPCRs were run using iTaq™ Universal SYBR Green Supermix (Bio-Rad) on a QuantStudio 3 Real-Time PCR System (Thermo Fisher Scientific, Waltham, MA, USA). The primers used are listed in Supplementary Table 1. The glyceraldehyde-3-phosphate dehydrogenase gene was used as a reference gene for normalization, and relative fold changes were calculated using the 2^−ΔΔCt^ method.

### Data Accession Number

The raw transcriptome data have been deposited in the NCBI Gene Expression Omnibus (GEO) database under accession no. GSE174377.

## Results

### Quality Control and Transcriptome Assembly

A raw dataset consisting of 360.7 million reads (~53.2 Gbps) was obtained through RNA-seq of the six samples. After filtering out the low-quality and ambiguous reads and trimming off the adaptor sequences, 60.2 (99.11%), 61.6 (98.97%), and 65.4 (99.03%) million high-quality clean reads from the control groups and 56.9 (98.99%), 58.0 (99.11%), and 58.6 (98.92%) million high-quality clean reads from the deoxyshikonin treatment groups were retained and used for assembly and analysis. The average Q30 value of all clean reads for the control and treatment groups were 91.54 and 91.44%, respectively, indicating that the clean reads were of high quality. Using the pig reference genome Sscrofa11, 92.56 and 90.92% of the total reads for the control and deoxyshikonin groups were uniquely mapped to the *Sus scrofa* genome, respectively, and the GC contents for the control and deoxyshikonin groups were 51.25 and 51.12%, respectively (Supplementary Table 2).

**Figure 1:**
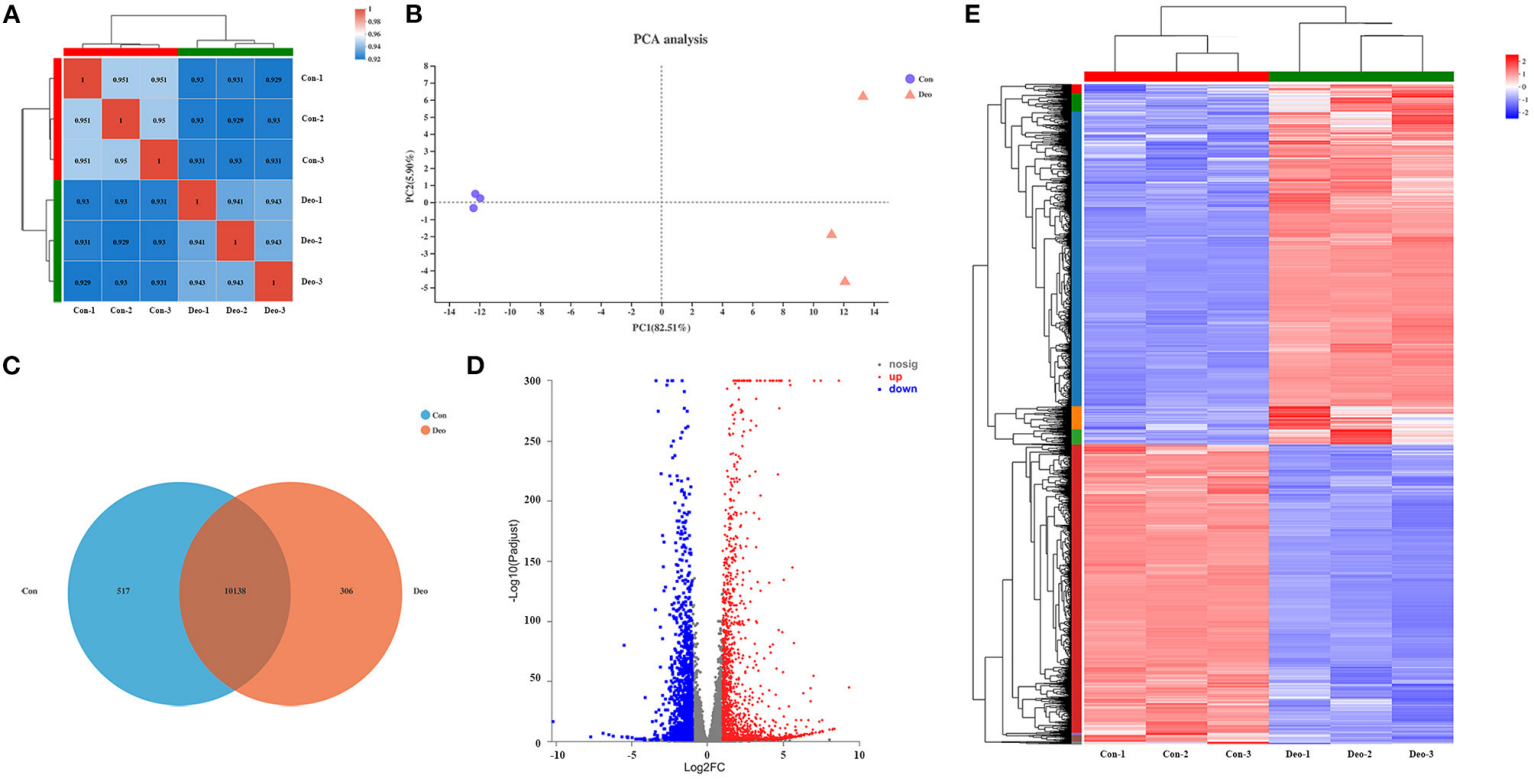
Correlation analysis and principal component analysis (PCA) of samples and identification of deoxyshikonin-unique genes and differentially expressed genes (DEGs). **(A)** Hierarchical clustering heatmap of the six samples using the Spearman method. R^2^ values represent the correlation between the expression profiles among the samples are presented in bold. **(B)** PCA plot of the samples. Each point represents one sample. Percentages are contribution ratios. **(C)** Venn diagram of referenced genes shared between the control and deoxyshikonin treatment groups. **(D)** Volcano plot of DEGs between the control and deoxyshikonin treatment groups. The X-axis represents the fold change of expression of DEGs and the Y-axis represents the statistical significance of the fold change. Red dots represent significantly upregulated DEGs, blue dots represent significantly downregulated genes, gray dots represent insignificant DEGs. **(E)** Clustering heatmap of the DEGs. The log_2_FC of gene abundance is indicated. Red and blue indicate high and low expression, respectively.

A sequencing saturation curve revealed that most genes with medium to high expression (FPKM > 3.5) were nearly saturated (ordinate tending to 1) at 40% of the mapped reads, which indicated that most expressed genes were covered (Supplementary Table 3, Supplementary Figure 1). Spearman's correlation matrix analysis showed that the correlation indices of the mapped genes were obviously different between the two treatments, whereas within-treatment differences were small, indicating a high consistency of measurements within each group and high reproducibility of the sequencing data (Figure 1A). In principal component analysis (PCA), principal components PC1 and PC2 explained 82.51 and 5.90%, respectively, of the distribution in the groups (Figure 1B). Deoxyshikonin-treated samples clustered together and were clearly separated from the control samples, suggesting that deoxyshikonin treatment substantially affected overall gene expression.

### Gene Expression Analysis and Functional Annotation

In total, 32,518 genes were detected in all samples, including 31,908 (98.12%) referenced genes and 610 (1.88%) unreferenced, novel genes. Of the 31,908 referenced genes, 27,521 genes could be annotated using the GO (23,249, 72.86%), KEGG (16,466, 51.60%), COG (22,660, 71.02%), NR (27,468, 86.08%), Swiss-Prot (23,826, 74.67%), and Pfam (19,407, 60.82%) databases (Supplementary Table 4). Among the 610 novel genes, 415 (68.03%) genes were successfully annotated using BLAST, including 171 genes (28.03%) in the GO database, 194 genes (31.80%) in the KEGG database, 187 genes (30.66%) in the COG database, 411 genes (67.38%) in the NR database, 162 genes (26.56%) in the Swiss-Prot database, and 118 genes (19.34%) in the Pfam database (Supplementary Table 4).

### Identification of Deoxyshikonin-Unique Genes and Functional Analysis

In total, 306 referenced genes with expression levels above 1 were uniquely present in the deoxyshikonin treatment group as indicated by the Venn diagram shown in Figure 1C (Supplementary Table 5). Among them, 250 genes could be assigned to 19 COG functional categories (Supplementary Figure 2A), including “intracellular trafficking, secretion, and vesicular transport” (Class U; 34 genes), “transcription” (Class K; 22 genes), and “posttranslational modification, protein turnover, chaperones” (Class O; 19 genes). GO analysis revealed that 243 deoxyshikonin-unique genes were annotated to 46 GO terms, mainly including “cell part” (173 genes), “cellular process” (155 genes), and “binding” (146 genes) (Supplementary Figure 2B). Seventy-one of the 306 deoxyshikonin-unique genes were assigned to 222 KEGG secondary categories, mainly including “signal transduction” (27 genes), “infectious diseases: viral” (22 genes), “cancer: overview” (21 genes), and “immune system” (19 genes) (Supplementary Figure 2C). According to GO enrichment analysis, the deoxyshikonin-unique genes were mostly enriched in regulatory and negative regulatory processes. KEGG enrichment analysis showed that arachidonic acid metabolism, breast cancer, and viral carcinogenesis were the most gene-enriched pathways.

Among the deoxyshikonin-unique genes, genes encoding the vitamin D receptor (VDR) were found, and their expression was increased (log_2_FC = 2.614) by deoxyshikonin treatment. The VDR regulates the expression of numerous genes (12) and is a valid target for modulating HDP expression via histone acetylation (13). Whether deoxyshikonin-induced HDP expression is regulated by the VDR requires verification. Furthermore, key genes in the MAPKKK8 immunomodulatory pathway and its downstream vital transcription factors FOBS, REL, and RELB were identified among the deoxyshikonin-unique genes, indicating the potential immunomodulatory effects of deoxyshikonin in pigs.

### Analysis of DEGs

DEGs after deoxyshikonin treatment were identified based on adjusted *P* < 0.05 and |log_2_ FC| ≥ 1. The DEGs identified, along with their fold changes and annotations, are presented in Supplementary Table 6. We identified 3,071 DEGs, including 1,677 upregulated and 1,394 downregulated genes (Figure 1D, Supplementary Table 6). A hierarchical clustering heatmap showing the expression profiles of the DEGs after deoxyshikonin treatment is presented in Figure 1E. The 1,677 upregulated genes included 110 genes with log_2_FC ≥ 5, 288 genes with 5 > log_2_FC ≥ 3, 285 genes with 3 > log_2_FC ≥ 2, and 995 genes with 2 > log_2_FC ≥ 1. Among the 1,394 downregulated genes, there were 11 genes with log_2_FC < −5, 33 genes with −5 ≤ log_2_FC < −3, 192 genes with −3 ≤ log_2_FC < −2, and 1,158 genes with −2 ≤ log_2_FC < −1.

**Figure 2:**
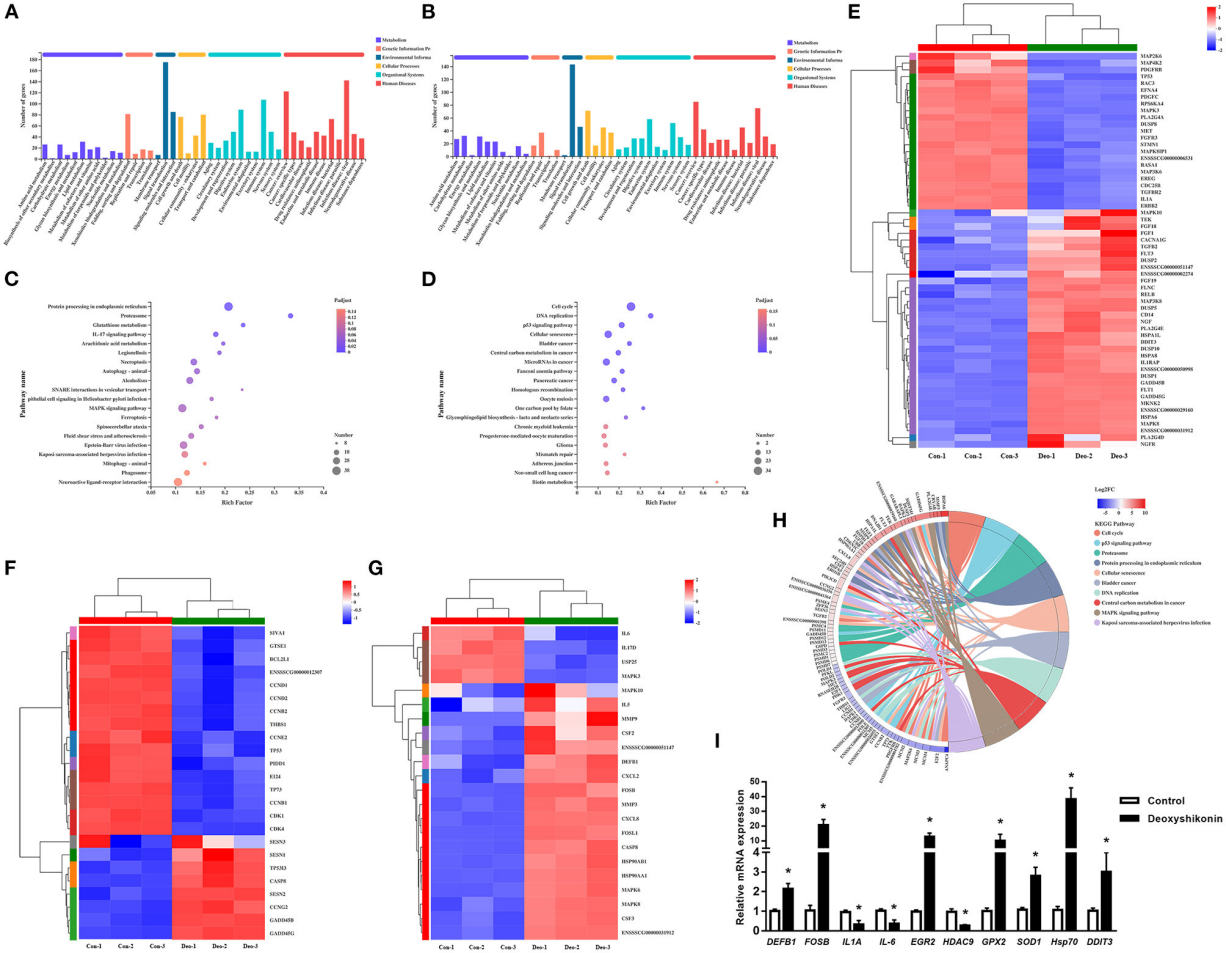
Kyoto Encyclopedia of Genes and Genomes (KEGG) annotations and enrichment analysis of differentially expressed genes (DEGs). **(A)** KEGG pathway annotations of upregulated DEGs. **(B)** KEGG pathway annotations of downregulated DEGs. **(C)** KEGG enrichment analysis of upregulated DEGs. **(D)** KEGG enrichment analysis of downregulated DEGs. Enrichment bubble chart showing KEGG pathway enrichment. The X-axis represents the enrichment ratio and the Y-axis represents the top 20 KEGG pathways. Number: bubble size representing the number of genes annotated to a KEGG pathway. P_adjust_: color indicates the enriched adjusted *P*-value. **(E)** Heatmap of DEGs enriched in the MAPK KEGG pathways, showing their expression profiles in the control and deoxyshikonin treatment groups. **(F)** Heatmap of DEGs enriched in the p53 KEGG pathways, showing their expression profiles in the control and deoxyshikonin treatment groups. **(G)** Heatmap of DEGs enriched in the IL17 KEGG pathways, showing their expression profile in the control and deoxyshikonin treatment groups. **(H)** KEGG chord plot of top 10 ranked KEGG pathways. Chords indicate a detailed relationship between the expression levels of DEGs (left semicircle perimeter) and their enriched KEGG pathways (right semicircle perimeter). The genes are linked to their annotated terms by colored ribbons. **(I)** Reverse-Transcription Quantitative PCR (RT-qPCR) of selected DEGs. Values represent means ± SEs, ^*^P < 0.05.

Among the DEGs, deoxyshikonin treatment significantly induced the defensin, beta 1 gene (DEFB1), with log_2_FC = 1.154. RT-qPCR confirmed that *DEFB1* expression was upregulated after deoxyshikonin treatment (Figure 2I). No other HDPs were found among the DEGs. Genes encoding downstream transcription factors of several important immunomodulatory pathways, including the activator protein-1 (AP1)-related genes, *FOSB* (log_2_FC = 5.051) and *FOSL1* (log_2_FC = 1.703), and the NF-κB-related genes, *REL* (log_2_FC = 1.484) and *RELB* (log_2_FC = 2.749), were significantly increased by deoxyshikonin treatment. Some anti-inflammatory and pro-inflammatory cytokine genes, including *IL-1A, IL-5, IL-6, IL-11, IL10RA*, and *IL17D*, were also significantly regulated by deoxyshikonin. The early growth response (EGR) family of transcription factors modulates immune response activation and proliferative responses (14). Deoxyshikonin significantly increased the expression of *EGR2* and *EGR3*, which play crucial roles in maintaining immune homeostasis (15). These results indicated that deoxyshikonin regulates immune functions.

Deoxyshikonin treatment significantly decreased histone deacetylase 9 gene (*HDAC9*) expression (log_2_FC = −2.058), whereas it upregulated the lysine demethylase genes *KDM2A* (log_2_FC = 1.374) and *KDM5A* (log_2_FC = 1.107). Maintenance of a histone hyperacetylation and hypomethylation status of target gene promoters largely contributes to transcript expression (16, 17). Several nutrients and natural products upregulate HDP expression through histone modification (7, 13). However, whether deoxyshikonin induces the expression of porcine HDP genes and other immunoregulatory genes through this way requires further investigation.

Deoxyshikonin is one of the main medicinal ingredients of *Lithospermi Radix*, a well-known traditional Chinese medicinal herb. *Lithospermi Radix* exerts strong antioxidant effects by exhibiting concentration-dependent 1,1-diphenyl-2-picrylhydrazyl radical-scavenging activity (5). Deoxyshikonin significantly promoted the gene expression of antioxidant defense-related glutathione peroxidase (*GPX2*, log_2_FC = 2.674 and *GPX3*, log_2_FC = 2.441), superoxide dismutase 1 (*SOD1*, log_2_FC = 1.063), thioredoxin-like 4 B (*TXNL4B*, log_2_FC = 1.761), P450 (cytochrome) oxidoreductase (*POR*, log_2_FC = 1.552), thioredoxin reductase 1 (*TXNRD1*, log_2_FC = 3.253), peroxiredoxin 1 (*PRDX1*, log_2_FC = 1.772), and thioredoxin-like 1 (*TXNL1*, log_2_FC = 1.558). RT-qPCR results showed that two cellular antioxidant genes (*GPX2* and *SOD1*) were upregulated by deoxyshikonin (Figure 2I). Together, these results indicated that deoxyshikonin has potential as an antioxidant against oxidizing free radicals.

Another noteworthy result is the upregulation of genes encoding heat shock proteins (HSPs) by deoxyshikonin. Eleven Hsp40 genes, 9 Hsp70 genes, and a few other high-molecular HSP genes were significantly induced after deoxyshikonin treatment. None of the HSPs was downregulated. HSPs are not only involved in protein folding and degradation as molecular chaperones, but also play potential roles in the innate immune system, resulting in resistance to pathogenic and viral infections (18). In particular, HSP70s have been demonstrated to be involved in the activation of several immune pathways (19). Shikonin has been demonstrated to induce HSP70, thus playing a cytoprotective role, in human lymphoma U937 cells (20). It will be interesting to study whether deoxyshikonin exerts its immune-regulatory functions or cytoprotective effects by activating HSPs.

### GO Functional Analysis of DEGs

Based on GO analysis, 2,745 genes among the 3,071 referenced DEGs were categorized into three principal GO terms of level 2 (Supplementary Figure 3A): molecular function (2,303 genes, 15 GO terms), cellular component (2,455 genes, 17 GO terms), and biological process (2,358 genes, 23 GO terms). The term “cell part” had the highest number of annotated DEGs (2,178 genes), followed by “cellular process” (1,920 genes) and “binding” (1,761 genes). The top 20 ranked GO terms of the DEGs are shown in Supplementary Figure 3B. The term “cellular response to topologically incorrect protein” under “biological process” was the most strongly enriched in DEGs. Twenty-one immune response-related GO terms were enriched in DEGs with *P* < 0.05, but adjusted *P* > 0.05. Among the top 20 ranked GO terms, “regulation of cell cycle phase transition” (73 genes) contained the most DEGs, followed by “cell division” (62 genes) and “negative regulation of cell cycle process” (58 genes).

### KEGG Pathway Analysis of DEGs

KEGG pathway analysis was carried out to investigate the biological functions of the DEGs. The 3,071 referenced DEGs, including 1,677 upregulated and 1,394 downregulated DEGs, were annotated to 335 KEGG secondary categories. The upregulated DEGs were mainly involved in “signal transduction” (175 genes) in environmental information, “infectious disease: viral” (142 genes) in human diseases, and “immune system” (107 genes) in organismal systems (Figure 2A). The downregulated DEGs were mainly involved in “signal transduction” (143 genes) in environmental information, “cancer: overview” (85 genes) in human diseases, and “cell growth and death” (71 genes) in cellular processes (Figure 2B).

KEGG enrichment analysis was conducted to identify DEGs in each signaling pathway. The DEGs were mostly enriched in “cell cycle,” “p53 signaling pathway,” and “proteasome,” whereas “pathways in cancer” and “MAPK signaling pathway” had the highest numbers of DEGs in the top 20 ranked pathways. The upregulated DEGs were mainly involved in “protein processing in endoplasmic reticulum,” “neuroactive ligand-receptor interaction,” and “MAPK signaling pathway,” which had the highest number of DEGs (Figure 2C). The downregulated DEGs were mainly involved in “cell cycle,” followed by “cellular senescence” and “microRNAs in cancer” (Figure 2D). DEGs mapped to enriched KEGG pathways are shown in Figure 2H.

Several immune response-related KEGG pathways were significantly enriched in DEGs. The MAPK pathway is a crucial signaling pathway that regulates the host innate immune response to infection and plays an important role in regulating pro-inflammatory and anti-inflammatory cytokines and chemokines (21). Thirty-five upregulated and 23 downregulated DEGs were assigned to the MAPK signaling pathway (adjusted *P* = 4.37E−2; Figure 2E). *MAPK10, MAPK8*, and *MAP3K8* were significantly increased in expression, whereas gene expression of *MAP3K6, MAPK8IP1, MAPK3, MAP4K2*, and *MAP2K6* was decreased. Several downstream transcription factor-related genes, such as *RELB* and *DDIT3*, were also induced by deoxyshikonin. The MAPK pathway is vital in the induction of defensin and cathelicidin expression by nutrients (1).

Tumor protein p53, a key tumor suppressor, is also involved in many other biological processes, including immune responses (22). The role of p53 in innate immune system regulation has been demonstrated (23). Activated p53 repressed human beta defensin 3 (hBD3) in primary oral keratinocytes and CaSki cells, whereas a reduction in cellular levels of p53 stimulated *hBD3* expression (24). In the present study, *TP53* expression was significantly suppressed (log_2_FC = −1.05) by deoxyshikonin treatment, indicating the potential role of p53 in the regulation of porcine HDPs. Twenty-four DEGs were assigned to the p53 signaling pathway (adjusted *P* = 1.63E−3; Figure 2F), mainly including cell cycle-associated and apoptosis-related genes, suggesting a regulatory effect of deoxyshikonin on the p53 pathway.

Among the enriched KEGG pathways, the IL-17 signaling pathway needs to be mentioned, despite the adjusted *P*-value of 0.11. The IL-17 signaling pathway included 22 DEGs, most of which (18 DEGs) were upregulated after deoxyshikonin treatment. IL-17 signaling contributes to the regulation of the innate and adaptive immune responses necessary for host defense (25). The IL-17 pathway is rapidly activated in response to infectious agents to recruit neutrophils and induce antimicrobial peptide production (26, 27). IL-17-regulated genes mainly include MAPKs, cytokines, and chemokines. Gene expression of *DEFB1* in the IL-17 signaling pathway was increased following deoxyshikonin treatment (Figure 2G).

## Data Availability Statement

The datasets presented in this study can be found in online repositories. The names of the repository/repositories and accession number(s) can be found at: https://www.ncbi.nlm.nih.gov/, GSE174377.

## Author Contributions

JW conceived the study and prepared the manuscript. WZ and XC performed the experiments. SW and YW performed the RNA extraction. WZ and HJ performed data analysis. All authors contributed to the article and approved the submitted version.

## Funding

This study was supported by the National Natural Science Foundation of China (Grant No. 31972576), the Beijing Natural Science Foundation (Grant No. 6202004), and the Special Program on Science and Technology Innovation Capacity Building of BAAFS (Grant No. KJCX201914).

## Conflict of Interest

The authors declare that the research was conducted in the absence of any commercial or financial relationships that could be construed as a potential conflict of interest.

## Publisher's Note

All claims expressed in this article are solely those of the authors and do not necessarily represent those of their affiliated organizations, or those of the publisher, the editors and the reviewers. Any product that may be evaluated in this article, or claim that may be made by its manufacturer, is not guaranteed or endorsed by the publisher.

## Supplementary Material

Supplementary Figure 1 Sequencing saturation curve for each sample. Each color line represents the saturation curve of gene expression at different expression levels in the sample.

Supplementary Figure 2 Functional annotation analysis of deoxyshikonin-unique genes based on the Clusters of Orthologous Groups (COG) **(A)**, Gene Ontology (GO) **(B)**, and Kyoto Encyclopedia of Genes and Genomes (KEGG) **(C)** databases.

Supplementary Figure 3 Gene Ontology (GO) annotations and enrichment analysis of differentially expressed genes (DEGs). **(A)** GO functional annotation of DEGs. **(B)** GO enrichment analysis of DEGs.

Supplementary Table 1 Primers used for Reverse-Transcription Quantitative PCR (RT-qPCR).

Supplementary Table 2 Statistics of filtered transcriptome data.

Supplementary Table 3 Statistics of reference genome comparison.

Supplementary Table 4 Functional annotation of genes and transcripts.

Supplementary Table 5 Annotation of deoxyshikonin-unique genes.

Supplementary Table 6 Annotation of differentially expressed genes (DEGs) between the control (Con) and deoxyshikonin-treated (Deo) groups.
