# Supplementary figures and images for: Deoxyshikonin-Induced Gene Expression Profile in Porcine Epithelial Cells
Source: Front Vet Sci. 2022 Jan 13;8:711721. doi: 10.3389/fvets.2021.711721 (PMC8792893; doi:10.3389/fvets.2021.711721)

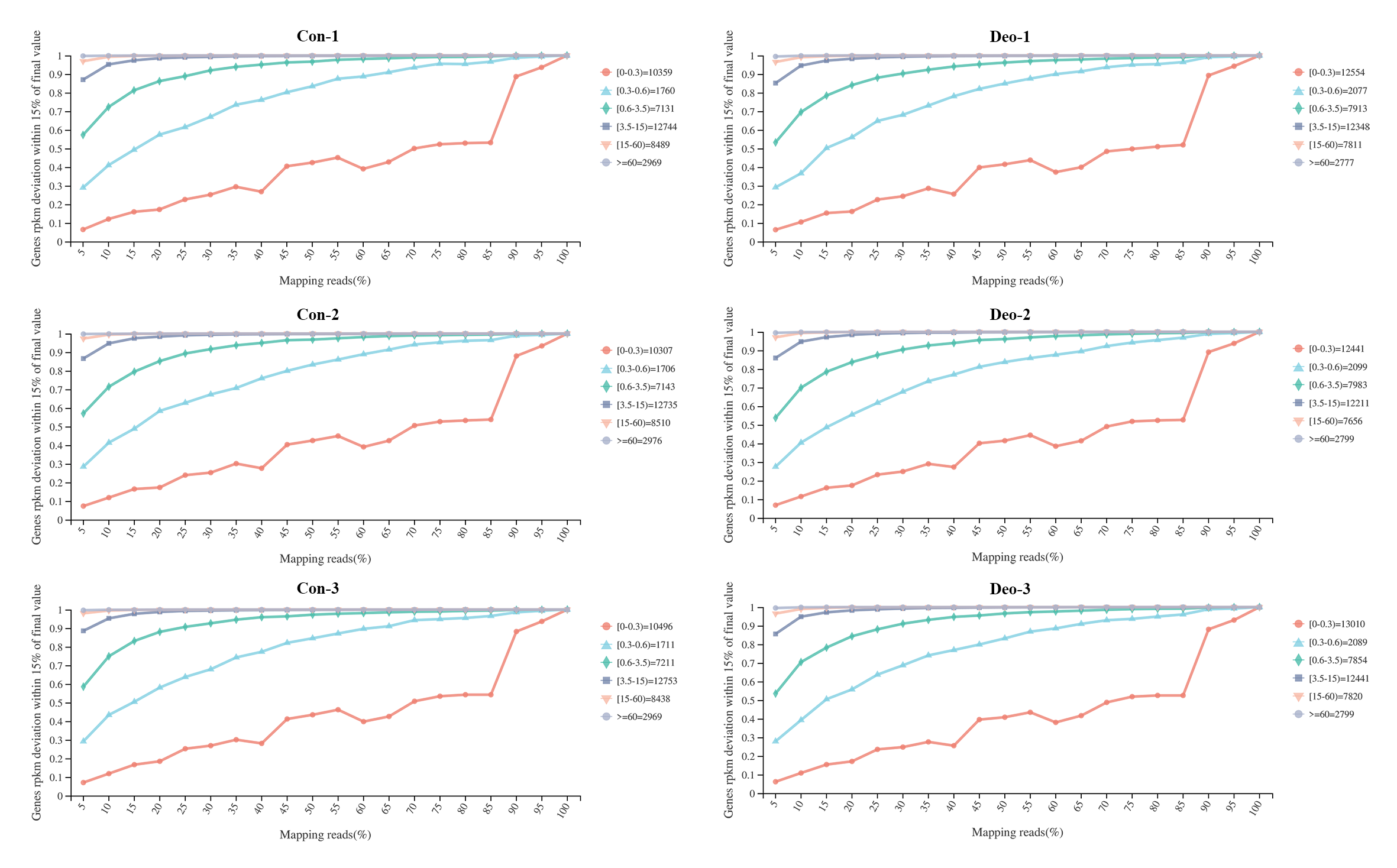

Supplement: Supplementary Figure 1 — Sequencing saturation curve for each sample. Each color line represents the saturation curve of gene expression at different expression levels in the sample. [file Image_1.TIF]

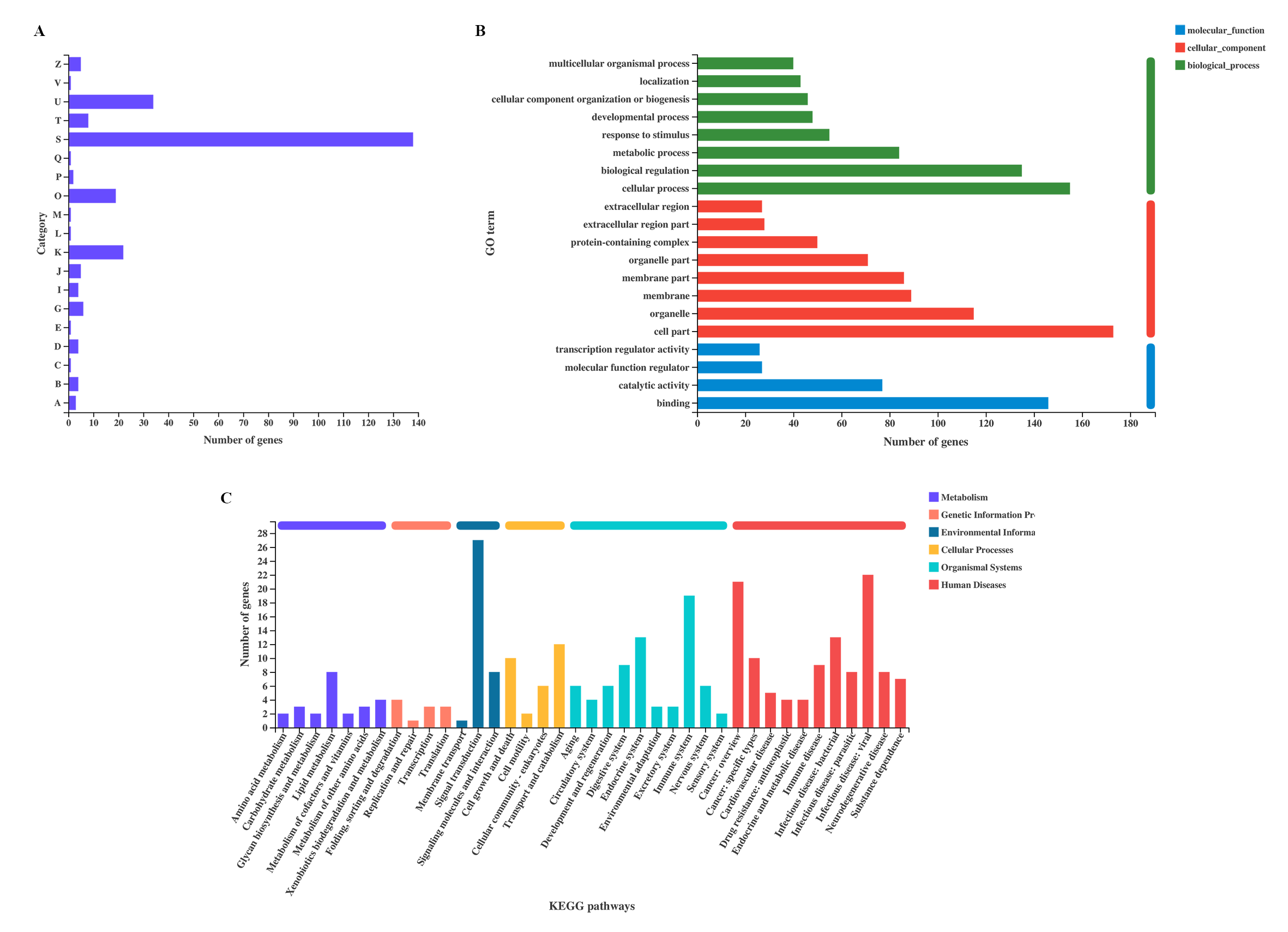

Supplement: Supplementary Figure 2 — Functional annotation analysis of deoxyshikonin-unique genes based on the Clusters of Orthologous Groups (COG) (A), Gene Ontology (GO) (B), and Kyoto Encyclopedia of Genes and Genomes (KEGG) (C) databases. [file Image_2.TIF]

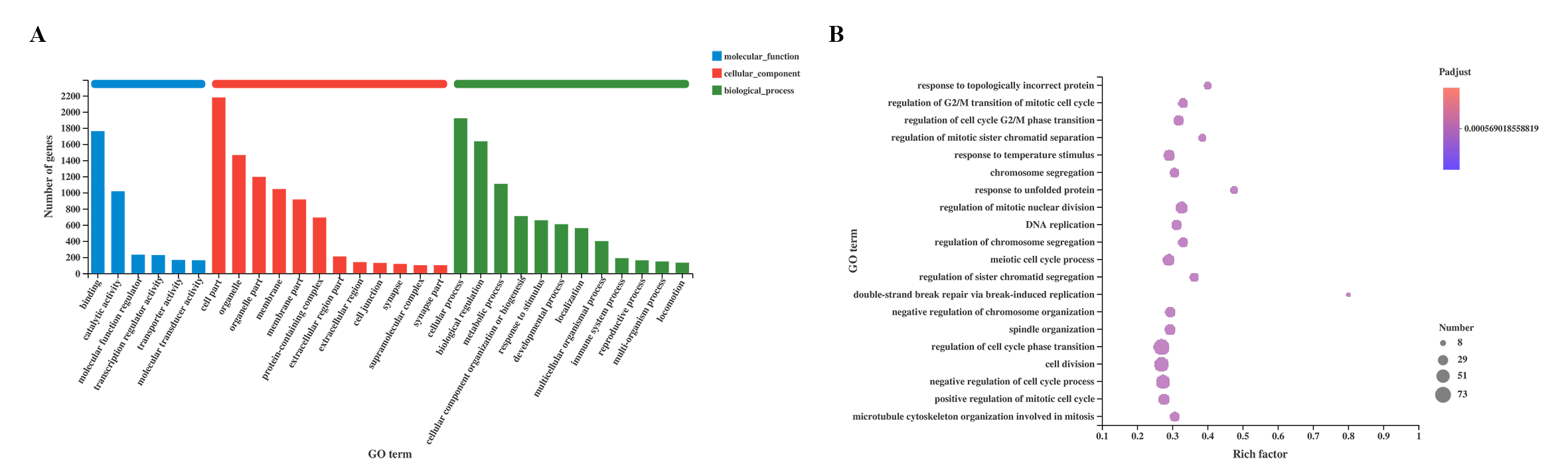

Supplement: Supplementary Figure 3 — Gene Ontology (GO) annotations and enrichment analysis of differentially expressed genes (DEGs). (A) GO functional annotation of DEGs. (B) GO enrichment analysis of DEGs. [file Image_3.TIF]
